# A Differential–Developmental Model (DDM): Mental Speed, Attention Lapses, and General Intelligence (*g*)

**DOI:** 10.3390/jintelligence5020025

**Published:** 2017-06-12

**Authors:** Thomas R. Coyle

**Affiliations:** Department of Psychology, University of Texas at San Antonio, San Antonio, TX 78249, USA; thomas.coyle@utsa.edu; Tel.: +1-210-458-7407; Fax: +1-210-458-5728

**Keywords:** cognitive development, differential–developmental model (DDM), Jensen’s box, intelligence, mental speed, attention lapses, reaction time, movement time

## Abstract

The aim of this paper is to provide a parsimonious account of developmental and individual differences in intelligence (measured as *g*). The paper proposes a Differential–Developmental Model (DDM), which focuses on factors common to intelligence and cognitive development (e.g., mental speed and attention lapses). It also proposes a complementary method based on Jensen’s box, a chronometric device. The device systematically varies task complexity, and separates two components of mental speed that differentially predict intelligence and cognitive development (reaction time and movement time). The paper reviews key assumptions of DDM, preliminary findings relevant to DDM, and future research on DDM.

## 1. Introduction

All models are wrong, but some are useful.

([1], p. 202)

The aim of this paper is to provide a parsimonious account of developmental and individual difference in intelligence (measured as *g*).^1^ To this end, the paper proposes a Differential–Developmental Model (DDM), which focuses on factors common to cognitive development and intelligence (e.g., mental speed and attention lapses). The model attempts to bridge the two disciplines at the heart of the Special Issue: differential psychology, which focuses on individual differences in intelligence, and cognitive development, which focuses on age differences in intelligence.

The paper highlights a complementary method based on Jensen’s paradigm ([3], pp. 27–32). The paradigm involves a chronometric device that systematically manipulates complexity (measured as bits of information). It also measures two components of mental speed (reaction time and movement time), which are differentially related to intelligence and cognitive development. Because the literature on developmental differences in mental speed is expansive, the paper will focus on the developmental period of childhood, with occasional references to aging. In addition, the paper will be anchored in intelligence research, with emphasis on research by Arthur Jensen [2,3]. Jensen was a titan in differential psychology. His research has important, but unexplored, implications for cognitive development, which will be discussed here.

Although the literature on mental speed is expansive, the starting point for the current paper is the voluminous research by Kail, who has focused on mental speed during childhood (e.g., ages 5 to 20 years), and Salthouse, who has focused on mental speed during normal aging (e.g., ages 20 to 80 years) (e.g., [4]; see also, [5,6]). Together, Kail and Salthouse have demonstrated that mental speed increases during childhood, decreases during aging, and contributes to higher order cognitions (e.g., [4]). In addition, they have shown that age-related differences on tests of mental speed (measuring different abilities) correlate with each other, suggesting the presence of a unitary speed factor, which is associated with higher order cognitions, including *g* (e.g., [7]).

As discussed below, DDM argues that age-related associations between mental speed and *g*-loaded constructs can be explained by an intervening variable after separating two components of speed. In particular, DDM identifies attention lapses as the mediator of speed–*g* relations, and emphasizes the need to separate reaction time and movement time, in order to obtain accurate estimates of the effects of speed.^2^

The paper contains five sections: (a) assumptions of DDM, (b) methods for testing DDM, (c) preliminary results relevant to DDM, (d) future research on DDM, and (e) implications and qualifications of DDM.

## 2. Assumptions of DDM

The central tenet of DDM is that cognitive processes unfold in real time and therefore are subject to chronometric analyses. Chronometric analyses measure temporal components of mental processes. The main chronometric measures are *mental speed*, measured by reaction times (RTs), and *variability in mental speed*, measured by trial-to-trial variability in RTs on elementary cognitive tasks (ECTs). ECTs are laboratory tasks that measure RTs to a reaction stimulus (RS), often a light, sound, or word. It will be argued that RTs and RT variability (along with attention lapses) can account for developmental and individual differences in intelligence.

DDM involves four key assumptions. The first is that mental processes become more efficient with increases in intelligence and age (in childhood). These increases in efficiency are associated with increases in mental speed, reflected by faster RTs, and decreases in variability, reflected by more consistent RTs. The more consistent RTs are related to fewer processing errors in the brain (e.g., fewer nerve cell communication errors) and in the mind (e.g., fewer attention lapses) ([8]; see also, [9,10]). Stated differently, the increases in efficiency result in more signal and less noise, which yields faster and more consistent RTs.

The second assumption is that mental speed should be partitioned into RT and movement time (MT). RT measures the speed of decision making before responding to the RS (e.g., light or sound). MT measures the speed of executing a response after responding to the RS (e.g., pushing a button). The two measures reflect different constructs, which should have different relations with intelligence. RT reflects a cognitive construct, which should be strongly associated with intelligence (another cognitive construct). In contrast, MT reflects a motor process, which should be weakly associated with intelligence. The RT–MT distinction is often ignored in chronometric research (cf. [11]), producing measurements with uncertain contributions of RT and MT, and yielding potentially inaccurate estimates of speed–intelligence relations.

The third assumption is that speed–intelligence relations are mediated by attention lapses (e.g., [8]; see also, [10]). In particular, DDM assumes that occasional lapses of attention are associated with slower and more variable responses to an RS. The lapses are assumed to be more frequent (and longer) at lower levels of intelligence and at younger ages in childhood, and to be associated with increases in cognitive processing errors, ultimately due to unreliable communication at the neural level.

The lapses of attention are attributed to a failure to maintain attention to a task goal, which is an aspect of attentional control (e.g., [10]). Attention lapses can be inferred from laboratory tasks such as the Stroop, flanker, antisaccade, and other tasks that require sustained attention to a task goal, particularly when the goal conflicts with a learned response.^3^ For example, in the Stroop, participants must keep in mind the goal of naming the color in which color names are printed, even when the color and word are in conflict. Such conflicts can produce a loss of goal maintenance (due to a lapse in attention), which is associated with naming errors or slow RTs.

The fourth assumption is that mental speed is associated with brain morphology, notably white matter integrity. White matter facilitates the propagation of nerve impulses and is related to neural and cognitive processing speed [13,14]. White matter development occurs over the first few decades of life, ending with the associative and frontal regions [15]. In general, age-related increases in white matter integrity are associated with increases in the speed and consistency of RTs, presumably because white matter integrity facilitates the speed and consistency of neural impulses between brain regions associated with cognitive performance (for a similar argument, see [7]).

**Figure 1:**
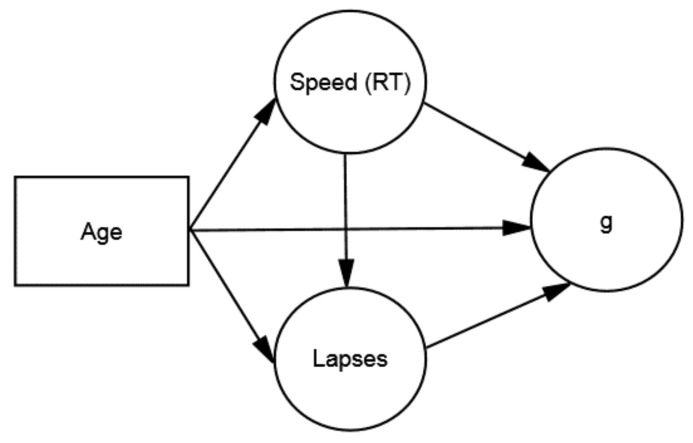
Structural model of age and cognitive constructs in Differential–Developmental Model (DDM).

Figure 1 depicts a structural model of the cognitive constructs in DDM. The model involves age and three factors: mental speed (RT), attention lapses, and intelligence (*g*). Using mediation terminology, RT is the causal factor, attention lapses are the mediator, and *g* is the criterion.^4^ Consistent with DDM, age should have direct effects on *g*, attention lapses, and RT, which should have a direct effect on lapses. In addition, individual differences in RT–*g* relations should be mediated by attention lapses, which are associated with faster (and more consistent) RTs and improvements on *g*-loaded tests.^5^ If attention lapses fully mediate RT–*g* relations, the causal path from RT to *g* should be non-significant, indicating that speed has no direct effect on intelligence, but derives its impact on intelligence through attention lapses. In addition, DDM predicts that age-related changes in white matter integrity (not shown) should be associated with faster RTs, fewer lapses, and higher levels of *g*. In contrast, age-related changes in MT, which reflect motor processing, should be negligibly associated with attention lapses or *g*, which reflects cognitive processing.

DDM differs from models that do not distinguish between RT and MT (e.g., [5,6]) and may therefore estimate speed–intelligence relations inaccurately (for a similar argument, see [11]). DDM also differs from models that regard other constructs as a basal requirement for more complex cognitions (e.g., fluid intelligence), or as a mediator of speed–intelligence relations. One such model is the cascade model of development [16]. Cascade models assume that age-related increases in speed lead to increases in working memory, which mediates speed–intelligence relations. In contrast to such models, DDM treats working memory as another manifestation of *g*, the criterion of the model. Indeed, the criterion in DDM is *any g*-loaded measure, which includes working memory, inductive reasoning, or fluid intelligence. Because all cognitive abilities are loaded with *g* (to some extent), a key issue is the degree of *g* saturation of the criterion, with stronger effects predicted for more complex criteria, which have higher *g* loadings ([18]; see also, [3], pp. 164–166). Finally, DDM differs from Jensen’s model [19], which focuses on individual differences in intelligence. In contrast to Jensen’s model, DDM focuses on *developmental differences* in mental speed and considers the mediating role of attention lapses on speed–intelligence relations.

## 3. Chronometric Methods, Jensen’s Box, and DDM

The predictions of DDM require chronometric methods that measure the speed and variability of RTs. The methods are illustrated here with Jensen’s box ([3], Figure 2.10, pp. 27–32; see also, [20]), which measures RTs and their relation to intelligence. Jensen’s box includes a home button surrounded by a semicircle of eight response buttons, arranged equidistantly around the home button. A trial begins with subjects pressing the home button and waiting for one of the eight response buttons to light up. After seeing a lit response button, subjects have to lift their finger from the home button and press the lit response button as fast as possible. Subjects complete multiple trials of the task, which measures two components of speed: RT and MT. RT measures the interval between the lighting of the response button and the release of the home button. MT measures the interval between the release of the home button and pressing the response button.

The procedure just described illustrates choice RT (CRT), in which subjects choose among eight response buttons. There are two other RT tasks. In simple reaction time (SRT), subjects see only one of the eight response buttons (the others are covered), and have to press the button when it is lit. In odd-man-out (OMO), subjects see all eight buttons and three of them light up simultaneously, with one button (the odd-man) being farther apart from the other two. Subjects have to press the odd-man while ignoring the other two. The three tasks (SRT, CRT, OMO) vary in complexity. SRT is the least complex (one button); CRT is moderately complex (eight buttons); and OMO is most complex (eight buttons, with discrimination instructions). On all tasks, the measures are RT, which reflects speed of decision making (initiating a response), and MT, which reflects motor speed (after initiating a response). The variability measures are standard deviation in RT (RTSD), which measures variability in RT over trials; and standard deviation in MT (MTSD), which measures variability in MT over trials.

Jensen’s box has desirable features for developmental and intelligence research. First, the three RT tasks (SRT, CRT, OMO) can be used with individuals varying widely in age or ability, with no changes in stimuli or administration instructions. Indeed, the three tasks have been used with individuals aged 5 to 90 years, and with IQs under 70 to over 130. In contrast, standardized tests of development (e.g., Piagetian tests) or intelligence (e.g., IQ tests) are typically used with a specific age range (e.g., children or adults) or ability range (IQs up to 140), and cannot measure intelligence levels outside of the designated range. Second, chronometric measures (RT and MT) are measured on a ratio scale, with equal intervals and a meaningful zero point. Ratio scales allow proportional comparisons such as “individual X is twice as fast as individual Y”, at all levels of age or ability, which facilitates precise comparisons at different ages. Such comparisons are not possible with IQ scores, which are age-normed, lack a meaningful zero point, and do not permit proportional comparisons at all levels of age and ability.

Although the findings discussed below are based on Jensen’s paradigm, they would apply to any paradigm that incorporates key features of Jensen’s paradigm. The key features are (a) the separation of RT and MT, and (b) the parametric manipulation of complexity. These features do not require access to the actual Jensen box and can be implemented with experimental software (e.g., E-Prime). For example, a keyboard could include a home button (spacebar) and response buttons (alphanumeric keys), which would allow for the separation of RT and MT. In addition, a monitor could present icons mirroring the keyboard configuration and varying in complexity (e.g., SRT, CRT, OMO). Indeed, with ubiquitous touch screen technology, Jensen’s paradigm could be performed entirely on a touch screen, with icons representing the buttons on Jensen’s box.

As discussed below, RT and MT are differentially associated with age and intelligence (RT is a stronger correlate), and therefore separation of RT and MT is needed to increase prediction accuracy. In contrast to Jensen’s paradigm, other speed paradigms—notably those using paper-and-pencil tasks—fail to separate RT and MT, and therefore may yield biased results. Another key feature of Jensen’s paradigm is the parametric manipulation of complexity, defined as the amount of information (i.e., bits) that must be processed before making a decision. Jensen’s paradigm can manipulate complexity by gradually increasing processing load in SRT and CRT tasks with 1, 2, 4, and 8 response alternatives.^6^ Such parametric manipulation controls for confounds (stimulus type and response modality), while gradually increasing processing load and complexity, which is a strong correlate of age and intelligence (e.g., [3], pp. 164–166; see also, [18]). In contrast to Jensen’s paradigm, other RT paradigms fail to control for complexity, or have unknown levels of complexity (e.g., paper-and-pencil tasks), and therefore cannot systematically examine the effects of complexity on age and intelligence.^7^

## 4. Preliminary Findings

### 4.1. Individual Differences in Intelligence

Jensen’s box has been widely used to examine individual differences in intelligence, notably IQ and other *g*-loaded measures ([3], pp. 155–186; see also, [19,23]). Consistent with DDM, RTs are faster and less variable at higher levels of intelligence, indicating that brighter people respond more consistently over time, a pattern associated with infrequent attention lapses. In addition, RTs predict intelligence better than MTs do, suggesting that differences in intelligence are attributable to the speed of initiating a response (i.e., deciding to respond), rather than the speed of executing a response (after deciding to respond). The RT–MT difference suggests that intelligence differences are attributable to cognitive processes in the brain, rather than motor processes in the periphery. Such a suggestion has developmental implications, since certain regions of the brain (e.g., the frontal lobes) are central for processing complex tasks, but are not fully developed until early adulthood [14], which suggests that the RT–MT difference may vary with development in childhood.

Finally, the RT–MT difference increases as a function of task complexity, measured by the three RT tasks ([3], pp. 155–186; see also, [19,23]). RTs are slower and more variable as task complexity increases (from SRT to CRT to OMO), while MTs show less pronounced slowing and variability with task complexity. In addition, RT correlations with intelligence increase as task complexity increases, whereas MT correlations show negligible increases with complexity. The results suggest that intelligence reflects the ability to handle complexity, defined as the amount of information that must be processed before making a response. As discussed below, it may also be the case that cognitive development in childhood reflects the ability to handle complexity, with development being associated with the ability to process larger amounts of information.

### 4.2. Cognitive Development in Childhood and Aging

Jensen’s box has also been used to examine mental speed in childhood (up to 20 years), and in aging (20 to 80 years) ([3], pp. 75–94). The childhood results are consistent with the assumptions of DDM. In particular, RTs become faster and less variable from age 9 to 14 years, which is a pattern associated with infrequent attention lapses.^8^ In addition, age differences in RT speed and variability differ with task complexity, with differences being larger on more complex tasks (CRT) and smaller on simpler tasks (SRT). The pattern suggests that age differences reflect the ability to process greater amounts of complexity (bits of information on RT tasks), presumably because older children process more information per unit of time, allowing them to handle more complexity (for a similar theory, see [7]). In contrast to RT, MT speed and variability show little change over the same age period (9 to 14 years). In addition, regressions of MT speed and variability on age show almost no relationship with task complexity. The results suggest that, unlike age differences in RT, age differences in MT (a motor response) are not associated with task complexity.

The aging results (20 to 80 years) mirror the childhood results, except that RTs become slower and more variable with age ([3], pp. 95–125), which is a pattern associated with more frequent (and of longer duration) attention lapses in DDM. In addition, age differences in RT vary with task complexity, with the differences increasing from SRT to CRT tasks with 1, 2, 4, and 8 response alternatives, suggesting that aging reflects decreases in the ability to process complexity. In contrast, age differences in MT are almost nonexistent and show no relationship to task complexity, suggesting that MT is not sensitive to cognitive aging.

The aging results replicate on diverse cognitive tasks [3], with RTs becoming slower and more variable (and MTs showing no change) from 20 to 80 years. The RT pattern has been obtained on tasks examining whether two words are synonyms or antonyms, whether two words are physically the same or different, and whether a probe digit was included in a previous string of digits. The similar results across different tasks suggest that aging differences in RT speed and variability reflect a global speed factor that contributes to general slowing in RTs across different tasks. The global speed factor has been observed across diverse tasks in adulthood (20 to 80 years; [6]) and in childhood (5 to 20 years; [5]).

### 4.3. The Worst Performance Rule (WPR)

DDM considers RT variability, defined as RTSD over trials. Consistent with DDM, RT variability shows reliable changes in development, decreasing in childhood and increasing in adulthood, with RT changes linked to attention lapses at different points in development.

RT variability is central to the worst performance rule (WPR), which predicts that the worst RTs (slowest RTs) reveal more about intelligence levels than the best RTs (fastest RTs) do. The WPR was discovered by Larson and Alderton [25], who ranked RTs obtained with young adults from fastest to slowest, and correlated the RTs with measures of intelligence. The RT correlations with intelligence increased steadily from fastest to slowest, indicating that the worst RTs were the best predictors of intelligence. In addition, ability differences were largest for the slowest (worst) RTs and smallest for the fastest (best) RTs. The pattern is consistent with the attention lapse assumptions of DDM. Compared to higher ability people, lower ability people experience longer duration attention lapses, which are associated with especially slow (worst) RTs. However, lower ability people sometimes notice the reaction stimulus (RS) at just the time it is presented, and therefore can respond to the RS as quickly as higher ability people do, making the fastest (best) RTs less sensitive to ability differences.

Coyle ([26]; see also, [8,27]) replicated the WPR with 8- to 10-year-olds who received five word-recall trials, and whose recall trials were ranked from best (most words recalled) to worst (fewest words recalled). Consistent with the WPR, the worst recall trials predicted IQ better than the best recall trials did, even after eliminating outliers, suggesting again that worst performance reveals more about intelligence than best performance does. In addition, IQ group differences (high vs. low) were larger for the worst recall trials than the best recall trials, which are less sensitive to ability differences.

This raises the question of whether or not there is a WPR for RT-age correlations during normal aging. Salthouse [28] examined this question by correlating RTs with age in 18- to 83-year-olds. RTs were binned by deciles, and RT-age correlations were computed within each decile. Contra the WPR, RT-age correlations decreased slightly from the fastest to slowest deciles ([28], Figures 2 and 5). In addition, the rate of RT slowing was similar across deciles for each age group, suggesting that RT slowing did not vary with age and RT decile ([28], Figure 1). The results are inconsistent with DDM, which assumes that increases in attention lapses over the lifespan should exacerbate age differences in slow (worst) RTs. Salthouse attributed the discrepancy between his study and other studies of the WPR to methodological differences in the RT tasks, the subjects’ ages, the psychometric tests, and the number of practice trials. Moreover, he did not separate MT and RT or systematically vary RT complexity, two factors that may contribute to the WPR, nor did he analyze narrower RT bins (i.e., bins smaller than deciles), which may have increased sensitivity and detected the WPR. Although Salthouse’s [28] study calls into question the generality of the WPR, methodological differences impede comparisons between his study and prior studies. To facilitate comparisons, future research should use a standardized set of RT tasks, such as those based on Jensen’s box, which separate RT and MT and systematically vary complexity.

## 5. Future Directions

In science, good models generate new and interesting research questions. This section will briefly describe three ideas for future research on DDM.

### 5.1. RT–MT Differences

DDM distinguishes between RT, which measures the speed of initiating a response, and MT, which measures the speed of executing a response (after initiation). Jensen’s box measures RT as the interval between RS presentation (lighting of response button) and response initiation (releasing the home button), and measures MT as the interval between response initiation and response completion (pressing the response button). The RT–MT distinction is important, because RT reflects the cognitive (decision-making) component of the response, which should be sensitive to attention lapses and age or intelligence differences, whereas MT reflects the motor component of the response, which should not be sensitive to attention lapses or age or intelligence differences.

The failure to account for RT–MT differences can distort chronometric findings on paper-and-pencil tasks designed to measure speed (for a similar view, see [11]). Such tasks include cross-out tasks, in which subjects see a target figure (a circle) and have to cross-out identical figures from a list, and matching tasks, in which subjects see a series of digits (3 9 2 8 5 9) and have to circle the digits that are the same (e.g., [29,30]; see also, [4,6]).^9^ A similar problem may exist on analogous computer-based matching tasks, in which subjects see a code table of digit-symbol pairs at the top of a screen and have to respond same or different to a probe pair below by pressing the appropriate key (e.g., [28], p. 155; see also, [6], p. 407). Such tasks are sensitive to development, with older children responding faster than younger children (and younger adults responding faster than elderly adults), and also to intelligence, with high IQ subjects responding faster than low IQ subjects. However, the age and IQ differences may be distorted by a failure to distinguish RT and MT (especially on paper-and-pencil tasks). In particular, based on DDM, age and IQ differences in responses may be contaminated by MT, which is less sensitive than RT to such differences and would depress age and IQ effects.

The failure to account for RT–MT differences can also distort the contributions of mental speed and working memory to intelligence and development [11]. Working memory holds and transforms information in immediate memory. It is measured with simple span tasks, which require manipulation of mental representations (e.g., backward digit span), and complex span tasks, which require holding information in mind while processing additional information. A complex span task includes sentence span, in which subjects read a series of sentences and then have to recall the last word in each sentence. The weight of evidence suggests that working memory mediates age-related effects of mental speed on intelligence, a pattern consistent with cascade models of development (e.g., [16,29]). Indeed, by some accounts, intelligence differences are almost entirely attributable to working memory, with a negligible effect of speed (e.g., [31,32]).

The finding that intelligence differences are mainly attributable to working memory (rather than speed) may be premature, for two reasons. First, the effects of speed on intelligence (relative to working memory) have been examined using paper-and-pencil or computerized tasks that do not separate RT and MT. As noted, the failure to separate RT and MT would yield a speed measure with an RT component, which is sensitive to intelligence differences, and a MT component, which is not sensitive to such differences. The upshot is that the MT component would depress the correlation of speed with intelligence, and the effects of speed would be underestimated, relative to working memory (for a similar argument, see [11]). (Although the argument focuses on intelligence differences, it would also apply to studies of age differences, which have ignored the MT–RT distinction (e.g., [30]). What is needed is a device, such as Jensen’s box, that separates RT and MT and isolates RT, which is sensitive to age and intelligence differences.

A second problem is that the effects of speed and working memory on intelligence are examined without controlling for task complexity, which is sensitive to intelligence differences [33]. Indeed, speed tasks often measure simple perceptual speed, such as the ability to identify simple stimuli (e.g., matching tasks), whereas working memory tasks often measure complex span, such as the ability to recall the last word in a series of sentences after reading the sentences (i.e., sentence span) (e.g., [32]; see also, [31]). The result is that speeded tasks are lower in complexity and weaker correlates of intelligence, whereas working memory tasks are higher in complexity and stronger correlates of intelligence. What is needed are tasks of speed and working memory that are equated in complexity (measured in bits), or methods that statistically control for complexity across tasks. Without such methods, lower-complexity speed tasks will (on average) yield weaker effects on intelligence, in comparison to higher-complexity working memory tasks.^10^

### 5.2. Reaction Stimulus (RS) Duration

DDM assumes that attention lapses vary with age and ability level. Attention lapses are assumed to be more frequent and longer for lower IQ subjects (compared to higher IQ subjects), younger children (compared to older children), and elderly adults (compared to younger adults). In addition, according to DDM, more frequent and longer lapses are associated with slower and more variable RTs, and produce the WPR, which predicts that the slowest RTs are the best indicators of intelligence. The prediction was supported by a correlational study of RTs on a sustained attention task, in which the slowest RTs correlated strongly with measures of intelligence and other abilities (e.g., working memory and executive control), whereas the fastest RTs correlated weakly with the same abilities ([10], Table 5). In addition, ability differences based on executive control (a correlate of intelligence) were largest for the slowest RTs and smallest for the fastest RTs ([10], Figure 3B), suggesting that longer lapses (associated with the slowest RTs), are the best predictors of cognitive ability (for a similar view, see [9]).

Correlational studies of DDM could be supplemented by experimental studies that manipulate the duration of the reaction stimulus (RS) using Jensen’s paradigm. In the standard paradigm, the RS lights up at a random interval (typically from 1 to 4 s after a preparatory beep), and it remains on until the subject presses the RS button, after which a new trial begins ([3], p. 29). A complementary approach would manipulate the duration of the RS, which would appear for a short (less than 250 ms) or long (more than 250 ms) duration. The short-duration condition would require more sustained attention to detect the fleeting RS, and therefore would be more sensitive to attention lapses. The long-duration condition would require less sustained attention because of the longer duration RS, and therefore would be less sensitive to attention lapses. Such a pattern has implications for the attention lapse assumptions of DDM. In particular, people who experience more frequent and longer lapses (lower IQ people, younger children, and elderly adults) would be expected to show lower levels of performance, especially in short-duration RS trials, whereas people who experience fewer and shorter lapses (higher IQ people, older children, and younger adults) would be expected to show higher levels of performance in all trials.

### 5.3. Fixed Interval Presentation of Reaction Stimulus (RS)

In the standard version of Jensen’s paradigm, the RS on each trial is presented during a random interval from 1 to 4 seconds after a preparatory stimulus (a beep). The random interval is designed to minimize preparatory responses to an RS presented at the same interval on each trial. An alternate approach would be to have the RS on each trial be presented during a fixed interval, perhaps two seconds after the preparatory beep. The fixed interval would allow subjects to anticipate the arrival of, and focus attention on, the impending RS, which would be presented at the same (fixed) interval. The fixed interval should facilitate focused attention, which would minimize the effects of attention lapses on RT. The effect of the fixed interval should differ for people who differ in attention lapses. In particular, the fixed interval should improve RTs for people with frequent lapses (lower IQ people, younger children, and elderly adults), who would benefit from a procedure that facilitates focused attention and minimizes the effects of the lapses. In contrast, the fixed interval should have less impact on people with less frequent lapses (higher IQ people, older children, and younger adults), who would benefit less from a procedure that minimizes the effects of the lapses.

## 6. Implications, Qualifications, and Open Questions

This section discusses implications, limitations, and future directions of DDM. It is based largely on comments raised by three anonymous reviewers. It discusses DDM and other theories, DDM and brain morphology, variability in speed–*g* relations, and speed–accuracy tradeoffs.

### 6.1. DDM and Other Theories

**Developmental theories.** DDM complements neo-Piagetian theories, which explain specific abilities and qualitative differences in thinking via general information processing mechanisms ([34], pp. 73–116). The information processing mechanisms may include mental speed and attention lapses (as in DDM), as well as executive functions and working memory [16]. Unlike neo-Piagetian theories, DDM proposes that all mental abilities are different manifestations of *g* and are influenced by mental speed and attention lapses. From this view, DDM is similar to theories, such as those proposed by Kail [5] and Salthouse [6], that place mental speed at the nexus of cognitive development.

DDM differs from other developmental theories of mental speed (e.g., [5,16]) in two ways. First, DDM argues that speed should be decomposed into RT, a strong correlate of intelligence, and MT, a weak correlate of intelligence. In contrast, other speed theories ignore the RT–MT distinction and may inaccurately estimate the influence of speed [11]. Second, DDM argues that attention lapses mediate speed–intelligence relations, whereas other theories focus on other mediators, such as working memory (e.g., [16]), which are different manifestations of *g* (the criterion predicted in DDM).

DDM can be compared to other models of development linking mental speed, working memory, and higher order cognition (e.g., fluid intelligence). One example is a developmental–differential model proposed by Demetriou et al. ([35]; see also, [36]), who describe relations among processing speed, working memory, and fluid intelligence from 4 to 16 years of age. Their model assumes that speed and working memory differentially index fluid intelligence in recurrent cycles during development, with speed being the leading indicator of fluid intelligence early in a cycle, and working memory being the leading indicator of fluid intelligence later in a cycle. Similar to Demetriou et al.’s [35] model, DDM emphasizes mental speed (but not working memory), and its association with age-related changes in higher order cognition. However, unlike Demetriou et al.’s [35] model, DDM is silent on cycles of development and their link to higher order cognition. Instead, DDM views the key mechanisms of development (speed and lapses) as being functionally equivalent throughout development, with faster RTs and fewer attention lapses being associated with higher levels of *g*, which subsumes working memory, fluid intelligence, and other *g*-loaded criteria.

**Diffusion models.** DDM complements diffusion models of RT [37,38]. Diffusion models assume that RTs reflect the rate of information accumulation until a response criterion is reached. The models describe two key parameters: drift rate, which measures the rate of information accumulation; and response criterion, which measures the amount of information needed before responding.^11^ Drift rate is assumed to index processing efficiency and the quality of evidence accumulated, while response criterion is assumed to index response caution. The two parameters can address speed–accuracy tradeoffs in RT. Higher drift rates reflect faster information accumulation, which is associated with faster responses and higher levels of *g* (e.g., working memory). Higher response criteria reflect more conservative response boundaries, which is associated with more accurate responding (e.g., [40]).

Diffusion models have been used to describe changes in RT during aging, with age-related slowing being attributed to more conservative response criteria (e.g., [41]). In addition, diffusion models may be able to explain RT-*g* relations without invoking attention lapses, which are central to DDM. One possibility is that interruptions of information accumulation may produce trial-to-trial variability in drift rate, which would be associated with slower and more variable RTs (and lower levels of *g*). Such a pattern would mimic the effects predicted by DDM, without invoking attention lapses (e.g., [40]). However, momentary lapses of attentional control may still contribute, in a top-down manner, to RT–*g* relations, a possibility that cannot be ruled out by diffusion models (cf. [40]). Such a possibility could be tested by measuring attention lapses (via Stroop, flanker, or other tasks) and evaluating their contribution to the development of RT–*g* relations using a diffusion model framework.

**Investment theories.** DDM has implications for investment theories of development (cf. [42]). Investment theories assume that more efficient processing increases specialization in specific domains (e.g., verbal or math). The specialization increases the influence of non-*g* factors related to specific abilities, and decreases the influence of *g* (variance common to all abilities) (for a similar argument, see [43]). In terms of DDM, more efficient processing would include faster mental speed and fewer attention lapses, which would facilitate learning in specific domains, leading to cognitive specialization. This prediction could be tested by examining the influence of *g* and non-*g* factors at different levels of speed. Consistent with investment theory, age-related increases in mental speed (which reflects more efficient processing) should increase cognitive specialization, which in turn would increase the influence of non-*g* factors and decrease the influence of *g*.

**Spearman’s Law of Diminishing Returns.** DDM is related to another theory with implications for cognitive specialization, Spearman’s Law of Diminishing Returns (SLODR) [44]. SLODR predicts that the *g* loadings of specific abilities decrease at higher levels of intelligence and age in childhood. The decrease in *g* loadings is assumed to reflect the influence of non-*g* factors at higher levels of age and ability and to be a consequence of cognitive specialization in specific domains (e.g., math or verbal) (e.g., [43]). In DDM, the influence of non-*g* factors may be attributable to age-related increases in mental speed (and decreases in attention lapses), which increases processing efficiency and facilitates cognitive specialization. This hypothesis could be tested by examining the effects of speed on the non-*g* residuals of specific abilities (obtained after removing *g*), which are assumed to reflect cognitive specialization (e.g., [45]).

### 6.2. DDM and Brain Morphology

DDM links mental speed to white matter integrity in the brain. White matter facilitates neural communication and is related to neural and cognitive processing speed [13,14], which is associated with attention lapses. Age-related increases in white matter integrity are associated with increases in the speed and consistency of RTs, presumably because white matter facilitates the speed and consistency of neural impulses between brain regions associated with cognition [13,14]. An open question is whether white matter integrity has stronger associations with RT than with MT, a prediction based on the assumption that white matter integrity is a marker of cognitive processing, which should be a stronger correlate of RT.^12^

### 6.3. DDM and Magnitude of Speed–Intelligence Relations

Estimates of speed–intelligence relations between a latent RT factor, based on multiple speed measures, and a latent ability (*g*) factor, based on multiple cognitive tests, range from 0.55 to 0.72, with an average of 0.62 ([3], pp. 171–172). However, the magnitude of the relations varies with different factors [3]. One is the measure of speed, with stronger relations for RT than for MT. Another is the complexity of the RT task, with stronger relations for more complex tasks (e.g., 8–button CRT) than for less complex tasks (e.g., 4–button CRT), a pattern consistent with the view that intelligence represents the ability to handle complexity [33]. A third factor is the *g*-loading (i.e., correlation with *g*) of the criterion, with stronger relations for tasks with higher *g* loadings. Forward and backward digit span represent tasks with different *g* loadings. Although both tasks involve recalling a series of digits, forward span requires recalling the digits in the presented order, whereas backward span requires reversing the order of the digits prior to recall. The additional process of reversing the digits constitutes an additional mental operation, which increases *g* saturation (and complexity) and speed–*g* relations (e.g., [18]; see also, [3], pp. 164–166).

### 6.4. DDM, Speed-Accuracy Tradeoffs, and RT-IQ Correlations

Speed–accuracy tradeoffs may explain RT–IQ correlations, which are central to DDM. One possibility is that high-IQ people respond quickly by sacrificing accuracy, which leads to more errors. Such sacrifices would yield positive relations between IQ and errors, and negative relations between RT and errors. Despite the plausibility of the pattern, RT–IQ data typically show the opposite pattern. IQ typically correlates negatively with errors (i.e., higher IQs predict fewer errors), and RT typically correlates positively with errors (i.e., slower RTs predict more errors) (e.g., [2], pp. 238–239). Moreover, high-IQ people typically respond faster at all levels of accuracy ([3], pp. 49–51), suggesting that RT–IQ relations cannot be attributed to response errors.

Speed–accuracy tradeoffs may also affect RT–age correlations, which may change during normal aging ([3], pp. 117–199). One possibility is that speed–accuracy preferences may shift during aging, with older people preferring more accurate responses at the expense of faster responses, a pattern that could be attributed to becoming less impulsive or more cautious. Such a pattern would yield greater accuracy at the expense of faster responding on cognitive tests, which are completed under time pressure. This possibility was examined by Phillips and Rabbit [46], who gave subjects age 50–79 years four timed cognitive tests, and then computed on each test an impulsivity measure, based on the difference between speed (items completed in time limit) and accuracy (items completed correctly). Although the impulsivity scores correlated positively with each other, suggesting a general impulsivity factor, the scores were unrelated to correct responses on the tests, or to age, suggesting that age deficits in cognitive tests cannot be attributed to changes in speed–accuracy preferences during aging. In addition, other research [47] indicates that although older people respond slowly on cognitive tasks, the mean difference in RTs between older and younger people is nearly identical (but reliably slower for older people), whereas the differences in response errors is negligible, suggesting that age differences in RT cannot be attributed to response errors.

### 6.5. Qualifications and Open Questions

DDM is a reductionist model of intelligence. Reductionist models have been criticized on the grounds that they do not account for the diverse abilities that comprise intelligence, such as the abilities described by hierarchical models of intelligence ([48]; but see, [49]). Hierarchical models include the Cattell–Horn–Carroll (CHC) model [50], which depicts *g* as a third-order factor, followed by broad abilities (e.g., fluid reasoning) and narrow abilities (e.g., quantitative reasoning). DDM takes no position on the number and types of specific abilities that comprise *g*, which depend on the number and types of cognitive tests used to model *g* ([2], pp. 85–88). However, DDM does make predictions about the effects of speed on *g*. In particular, consistent with SLODR, the influence of *g* is assumed to wane with increases in processing efficiency (reflected by faster RTs and fewer attention lapses) during childhood. The increases in processing efficiency are assumed to produce cognitive specialization in specific domains (e.g., verbal or math), which decreases the *g* loadings of specific abilities, and increases their non-*g* (residual) loadings.

DDM omits constructs included in other models of speed and development. One such construct is working memory, which holds and transforms information in immediate memory. Working memory is regarded as a basal requirement for more complex cognitions such as inductive reasoning [29] and fluid intelligence [16]. It is also regarded as a mediator in cascade models of cognitive development [16]. Cascade models assume that age-related increases in mental speed lead to increases in working memory, which lead to increases in fluid intelligence. In contrast to such models, DDM treats working memory, fluid intelligence, and other abilities as *g*-loaded criteria, which are predicted by mental speed and attention lapses. Indeed, DDM assumes that all abilities are merely manifestations of *g* that differ in their *g* loadings and levels of complexity. Moreover, DDM assumes that if different abilities have similar *g* loadings, those abilities will have similar associations with the other factors in the model.

## 7. Epilogue

The aim of this paper has been to provide a parsimonious account of developmental and individual differences in intelligence (measured as *g*). In particular, the paper describes DDM, a model involving constructs common to intelligence and age differences (e.g., mental speed and attention lapses). In addition, the paper highlights a chronometric paradigm proposed by Jensen ([3]; see also, [20]). The paradigm separates RT and MT, two components of speed that differentially contribute to intelligence. Future research should examine factors related to DDM (e.g., attention lapses, non-*g* factors, diffusion model parameters, white matter integrity), which may be differentially associated with developmental and individual differences in intelligence.

## Footnotes

1 In the current article, the term “intelligence” refers to general intelligence (*g*), which reflects variance common to cognitive tests. *g* reflects the fact that people who perform well on one cognitive test generally perform well on all other tests, yielding positive correlations among the tests. Tests that correlate strongly with *g* (i.e., *g*-loaded tests) include IQ tests, fluid reasoning tests (e.g., Raven’s Matrices and Cattell’s Culture Fair Test), and academic aptitude tests (e.g., SAT, ACT, and PSAT). *g* contributes strongly to a test’s predictive validity at school and work (e.g., [2], pp. 274–294).

2 It should be emphasized that, in DDM, the explanandum is the age-related association between mental speed (the causal factor) and *g* (the outcome). DDM proposes that the association between speed and *g* can be explained by attention lapses after separating two key components of speed (movement time and reaction time).

3 Other indicators of attention lapses may include eye movement latencies in eye tracking studies, a suggestion made by an anonymous reviewer. Indeed, in infancy, eye movement latencies following the presentation of a target stimulus predict later IQ (at 5 years), with faster latencies predicting higher IQs [12]. These latencies may index attention lapses linked to IQ. In particular, if high-IQ individuals experience fewer (and shorter duration) attention lapses, their latencies following a target stimulus should be shorter and less variable (cf. [12]). Eye movement latencies represent one indicator of attention lapses. Ideally, attention lapses and other DDM constructs should be modeled using latent variable analysis and multiple indicators, which would reduce measurement error (e.g., method variance) and increase variance related to key constructs (e.g., lapses, speed, and *g*).

4 It is worth noting that *g* (the criterion) is strongly associated with fluid intelligence, defined as general reasoning and problem-solving ability, and crystallized intelligence, defined as acquired knowledge and skills. For example, Jensen ([2], p. 126) estimated the average *g* loading of the Verbal and Performance scales of the Wechsler Intelligence Scale for Children (WISC). The Verbal and Performance subtests resemble crystallized and fluid types of tests, respectively. The average *g* loading of the two scales was 0.89 (a value close to the reliability of the WISC IQ), suggesting that fluid and crystallized abilities largely reflect *g*.

5 The model depicts paths from speed (RT) to lapses, which implies speed affects lapses, which in turn affect *g*. In particular, the model examines whether lapses mediate the link between speed and *g*, and whether the direct effect of speed on *g* becomes nonsignificant with lapses in the model. This pattern is consistent with developmental cascade models [16], which predict that lower order processes (e.g., mental speed) affect higher order processes (e.g., working memory), which in turn affect *g*. In addition, the pattern is consistent with theories suggesting that fast mental speed helps maintain goal representations and avoid attention lapses. An alternative model (not depicted) could reverse the direction of the path between speed and lapses. This model would examine whether speed mediates a link between lapses and *g*, a hypothesis consonant with theories that attention lapses represent lower order processes (perhaps reflecting neural noise) that affect speed, which in turn affects *g* (cf. [17]). Ultimately, the direction of the path depends on whether lapses are assumed to reflect basic processes (e.g., neural errors) that precede speed, or to reflect higher order cognition that follows speed. DDM depicts a pattern similar to that described by developmental cascade models (e.g., [16]), which assume that lower order processes (mental speed) predict higher order processes (attention lapses), which help maintain goal states during information processing.

6 A bit (binary digit) is a unit of information that can have only one of two values (commonly represented 0 or 1). In information theory, a bit is defined as the amount of information that reduces uncertainty by one-half. In Jensen’s paradigm, the set of response alternatives are normally 1, 2, 4, and 8 buttons, corresponding to 0, 1, 2, and 3 bits.

7 This article focuses on RT tasks, which require an overt response (a button release or press) following stimulus presentation. Another speeded task, which is not reviewed here, is inspection time (IT). IT measures speed of information intake, based on the shortest exposure duration required to detect a stimulus [21,22]. In a standard IT task, participants see two lines, one of which is longer than the other one, and are asked to identify which line is longer. IT does not require an overt response, which eliminates MT. However, IT does not parametrically vary complexity, making it unsuitable for studying complexity and intelligence. What is needed is an IT paradigm that systematically manipulates complexity. Such a paradigm would complement Jensen’s RT paradigm and allow the examination of relationships between intelligence and IT complexity.

8 Jensen’s paradigm has been used with 5- to 20-year-olds ([3], pp. 79–83). A reviewer wondered whether the paradigm could be used with children younger than 5 years of age. The answer is speculative. First, other RT paradigms with similar instructions (“identify the picture on the right”) have been used with children younger than 5 years [12], suggesting that younger children could perform Jensen’s paradigm. Second, increases in processing efficiency (via reductions in attention lapses) would be expected to yield faster RTs at any point in development, even in infancy. Indeed, stability in RTs has been observed from infancy to 5 years and has been linked to later IQ, presumably because faster RTs lead to more efficient information processing, which leads to better performance on IQ tests [12]. Finally, increases in processing efficiency are theoretically linked to increases in brain myelination, which undergoes rapid development in the first few years of life [24]. The development of myelination during this period would be expected to increase processing efficiency, which in turn would lead to faster and less variable RTs.

9 Some of these paper-and-pencil tests have been adapted for computer administration. For example, in a computer adaptation of the matching task, participants compare two stimuli (e.g., 8··8) and respond by pressing an “S” key for same, or a “D” key for different (e.g., [6], p. 407). Although the key presses in computer adaptations may reduce the influence of the motor component, they do not separate MT and RT and therefore cannot evaluate the relative contribution of each speed component.

10 An anonymous reviewer requested an example of a procedure to equate working memory and mental speed in complexity. Conceptually, such a procedure might involve performing a task analysis and (a) comparing tasks with similar levels of complexity (similar numbers of bits) or (b) comparing tasks with different levels of complexity after statistically controlling for complexity. A related approach might be to compare the influence of mental speed and working memory on the same task, which would effectively control for complexity. An example might be to use backward digit span (a working memory task) to measure rehearsal speed as an indicator of mental speed and items recalled as an indicator of working memory. In any case, the key issue is to perform a task analysis (a priori) to identify levels of complexity, which is needed to control for complexity.

11 Diffusion models also incorporate a non-decision parameter (*T*_er_), which reflects the speed of non-decision processes such as stimulus encoding, motor programming, and motor speed, which may influence MT. Unfortunately, because this non-decision parameter measures both input (e.g., stimulus encoding) and output (e.g., motor speed) processes, it cannot isolate MT or provide an unbiased estimate of motor speed (cf. [39]).

12 It should be noted that age-related trends in RT are typically nonlinear, decreasing exponentially during childhood (5- to 20-years) and increasing nonlinearly during normal aging (20- to 80-years) ([3], pp. 77–78; [5], Figures 2 and 3; cf. [6], Figure 1). An anonymous reviewer noted that these nonlinear trends in RT may track nonlinear trends in white matter integrity (e.g., [13,14]), which is associated with RT speed and variability. In particular, nonlinear increases in white matter integrity during childhood, culminating with the frontal lobes, should correlate with nonlinear decreases in RT and RT variability. In contrast, nonlinear decreases in white matter during aging should be associated with the opposite pattern.

## References

[1] Box G.E.P., Launer R.L., Wilkinson G.N. (1979). Robustness in the strategy of scientific model building. Robustness in Statistics.

[2] Jensen A.R. (1998). The g Factor: The Science of Mental Ability.

[3] Jensen A.R. (2006). Clocking the Mind: Mental Chronometry and Individual Differences.

[4] Kail R., Salthouse T.A. (1994). Processing speed as a mental capacity. Acta Psychol..

[5] Kail R. (1991). Developmental change in speed of processing during childhood and adolescence. Psychol. Bull..

[6] Salthouse T.A. (1996). The processing-speed theory of adult age differences in cognition. Psychol. Rev..

[7] Coyle T.R., Pillow D.R., Snyder A.C., Kochunov P. (2011). Processing speed mediates the development of general intelligence (*g*) in adolescence. Psychol. Sci..

[8] Coyle T.R. (2003). A review of the worst performance rule: Evidence, theory, and alternative hypotheses. Intelligence.

[9] McVay J.C., Kane M.J. (2012). Drifting from slow to “D’oh!”: Working memory capacity and mind wandering predict extreme reaction times and executive control errors. J. Exp. Psychol. Learn. Mem. Cognit..

[10] Unsworth N., Redick T.S., Lakey C.E., Young D.L. (2010). Lapses in sustained attention and their relation to executive control and fluid abilities: An individual differences investigation. Intelligence.

[11] Coyle T.R. (2013). Effects of processing speed on intelligence may be underestimated: Comment on Demetriou et al. (2013). Intelligence.

[12] Dougherty T.M., Haith M.M. (1997). Infant expectations and reaction time as predictors of childhood speed of processing and IQ. Dev. Psychol..

[13] Kochunov P., Coyle T., Lancaster J., Robin D.A., Hardies J., Kochunov V., Bartzokis G., Stanley J., Royall D., Schlosser A.E. (2010). Processing speed is correlated with cerebral health markers in the frontal lobes as quantified by neuro-imaging. Neuroimage.

[14] Miller E.M. (1994). Intelligence and brain myelination: A hypothesis. Pers. Individ. Differ..

[15] Fields R.D. (2010). Changes in the brain’s white matter. Science.

[16] Fry A., Hale S. (1996). Processing speed, working memory, and fluid intelligence: Evidence for developmental cascade. Psychol. Sci..

[17] Jensen A.R. (2011). The theory of intelligence and its measurement. Intelligence.

[18] Jensen A.R. (1992). Understanding *g* in terms of information processing. Educ. Psychol. Rev..

[19] Jensen A.R., Sternberg R.J., Pretz J.E. (2005). Mental chronometry and the unification of differential psychology. Cognition and Intelligence: Identifying the Mechanisms of the Mind.

[20] Hick W.E. (1952). On the rate of gain of information. Q. J. Exp. Psychol..

[21] Deary I.J., Stough C. (1996). Intelligence and inspection time: Achievements, prospects, and problems. Am. Psychol..

[22] Grudnik J.L., Kranzler J.H. (2001). Meta-analysis of the relationship between intelligence and inspection time. Intelligence.

[23] Jensen A.R., Eysenck H.J. (1982). Reaction time and psychometric *g*. A Model for Intelligence.

[24] Aubert-Broche B., Fonov V., Leppert I., Pike G.B., Collins D.L., Metaxas D., Axel L., Fichtinger G., Székely G. (2008). Human brain myelination from birth to 4.5 years. Medical Image Computing and Computer-Assisted Intervention—MICCAI 2008.

[25] Larson G.E., Alderton D.L. (1990). Reaction time variability and intelligence: A ‘‘worst performance’’ analysis of individual differences. Intelligence.

[26] Coyle T.R. (2001). IQ is related to the worst performance rule in a memory task involving children. Intelligence.

[27] Coyle T.R. (2003). IQ, the worst performance rule, and Spearman’s law: A reanalysis and extension. Intelligence.

[28] Salthouse T.A. (1998). Relations of successive percentiles of reaction time distributions to cognitive variables and adult age. Intelligence.

[29] Kail R.V. (2007). Longitudinal evidence that increases in processing speed and working memory enhance children’s reasoning. Child Dev..

[30] Kail R.V., Ferrer E. (2007). Processing speed in childhood and adolescence: Longitudinal models for examining developmental change. Child Dev..

[31] Colom R., Abad F.J., Quiroga M.A., Shih P.C., Flores-Mendoza C. (2008). Working memory and intelligence are highly related constructs, but why?. Intelligence.

[32] Colom R., Rebollo I., Palacios A., Juan-Espinosa M., Kyllonen P.C. (2004). Working memory is (almost) perfectly predicted by *g*. Intelligence.

[33] Gottfredson L.S. (1997). Why *g* matters: The complexity of everyday life. Intelligence.

[34] Bjorklund D.F. (2000). Children’s Thinking: Developmental Function and Individual Differences.

[35] Demetriou A., Spanoudis G., Shayer M., Mouyi A., Kazi S., Platsidou M. (2013). Cycles in speed-working memory-G relations: Towards a developmental-differential theory of mind. Intelligence.

[36] Tourva A., Spanoudis G., Demetriou A. (2016). Cognitive correlates of developing intelligence: The contribution of working memory, processing speed and attention. Intelligence.

[37] Ratcliff R. (1978). A theory of memory retrieval. Psychol. Rev..

[38] Ratcliff R., Schmiedek F., McKoon G. (2008). A diffusion model explanation of the worst performance rule for reaction time and IQ. Intelligence.

[39] Schubert A.-L., Frischkorn G.T., Hagemann D., Voss A. (2016). Trait characteristics of diffusion model parameters. J. Intell..

[40] Schmiedek F., Oberauer K., Wilhelm O., Süß H.-M., Wittmann W.W. (2007). Individual differences in components of reaction time distributions and their relations to working memory and intelligence. J. Exp. Psychol. Gen..

[41] Ratcliff R., Thapar A., McKoon G. (2011). Effects of aging and IQ on item and associative memory. J. Exp. Psychol. Gen..

[42] Cattell R.B. (1987). Intelligence: Its Structure, Growth and Action.

[43] Coyle T.R. (2015). Relations among general intelligence (*g*), aptitude tests, and GPA: Linear effects dominate. Intelligence.

[44] Spearman C. (1932). The Abilities of Man: Their Nature and Measurement.

[45] Coyle T.R., Purcell J.M., Snyder A.C., Kochunov P. (2013). Non-*g* residuals of the SAT and ACT predict specific abilities. Intelligence.

[46] Phillips L.H., Rabbitt P.M.A. (1995). Impulsivity and speed-accuracy strategies in intelligence test performance. Intelligence.

[47] Smith G.A., Bewer N. (1995). Slowness and age: Speed-accuracy mechanisms. Psychol. Aging.

[48] Ceci S.J. (1990). On the relation between microlevel processing efficiency and macrolevel measures of intelligence: Some arguments against current reductionism. Intelligence.

[49] Neubauer A.C., Bucik V. (1996). The mental speed-IQ relationship: Unitary or modular?. Intelligence.

[50] McGrew K.S. (2009). CHC theory and the human cognitive abilities project: Standing on the shoulders of the giants of psychometric intelligence research. Intelligence.

